# Plasma Exchange as an Adjunctive Therapeutic Option for Severe and Refractory Antineutrophil Cytoplasmic Antibody-Negative Microscopic Polyangiitis and Granulomatosis with Polyangiitis

**DOI:** 10.3390/medicina61122184

**Published:** 2025-12-09

**Authors:** Woongchan Rah, Oh Chan Kwon, Jang Woo Ha, Yong-Beom Park, Sang-Won Lee

**Affiliations:** 1 Division of Rheumatology, Department of Internal Medicine, Yonsei University College of Medicine, Seoul 03722, Republic of Korea; wcrah@yuhs.ac (W.R.);; 2 Division of Rheumatology, Department of Internal Medicine, Gangnam Severance Hospital, Yonsei University College of Medicine, Seoul 06273, Republic of Korea; ockwon@yuhs.ac; 3 Division of Rheumatology, Department of Internal Medicine, Yongin Severance Hospital, Yonsei University College of Medicine, Yongin 16995, Gyeonggi-do, Republic of Korea; hjwnmk@yuhs.ac; 4 Institute for Immunology and Immunological Diseases, Yonsei University College of Medicine, Seoul 03722, Republic of Korea

**Keywords:** microscopic polyangiitis, granulomatosis with polyangiitis, ANCA-negative, plasma exchange, prognosis

## Abstract

*Background and Objectives*: This study investigated and compared the efficacy of therapeutic plasma exchange (PEX) between antineutrophil cytoplasmic antibody (ANCA)-positive and ANCA-negative patients with microscopic polyangiitis (MPA) and granulomatosis with polyangiitis (GPA) presenting with diffuse alveolar haemorrhage (DAH) and rapidly progressive glomerulonephritis (RPGN). *Materials and Methods*: A total of 336 patients with ANCA-associated vasculitis were screened, and 34 patients with MPA/GPA receiving PEX for DAH or RPGN were included. PEX was performed a total of 5–6 times consecutively (three times a week × 2 weeks) in all 34 patients. All-cause mortality (ACM) and end-stage kidney disease (ESKD) were evaluated as poor outcomes of MPA/GPA. Clinical data and poor outcomes were compared between ANCA-positive and ANCA-negative MPA/GPA patients receiving PEX. *Results*: The median age of the 34 MPA/GPA patients was 67 years (15 men and 19 women), of whom two were diagnosed with ANCA-negative vasculitis. Among the 34 patients, 28 (82.4%) received PEX owing to RPGN, and 6 (17.6%) due to DAH. During follow-up, 13 patients (38.2%) died, and 15 (44.1%) progressed to ESKD. Serum protein and C-reactive protein levels at AAV diagnosis were higher in ANCA-positive MPA/GPA patients than in ANCA-negative patients, although the difference was not statistically significant. Similarly, there were no differences in ACM or ESKD between the two groups during follow-up. Survival analysis showed that ANCA-positive MPA/GPA patients did not have significantly different cumulative patient or ESKD-free survival rates compared to ANCA-negative patients. *Conclusions*: This pilot study is the first to demonstrate the clinical feasibility of PEX in managing severe and refractory ANCA-negative MPA and GPA.

## 1. Introduction

Microscopic polyangiitis (MPA) and granulomatosis with polyangiitis (GPA) are subtypes of antineutrophil cytoplasmic antibody (ANCA)-associated vasculitis (AAV). These vasculitides are characterised by the pathologically typical findings of fibrinoid necrotising vasculitis involving capillaries, arterioles, and venules [1,2]. While various methods have been introduced recently to minimise the risks of invasive biopsy and to assess the inflammatory burden at the capillary level, such as nailfold microscopy and optical coherence tomography angiography [3,4], the classification of MPA/GPA still relies on the presence of ANCA and clinical features [5,6,7]. In terms of clinical features, MPA and GPA can be distinguished by differences in the distribution of major organs involved and their associated manifestations, as well as by their histological features. MPA predominantly presents with renal and pulmonary involvement, whereas GPA mainly affects the upper and lower respiratory tracts [5,6,7]. In terms of treatment, the guidelines proposed by the American College of Rheumatology (ACR) and the European Alliance of Associations for Rheumatology (EULAR) currently recommend rituximab (RTX) or cyclophosphamide (CYC) as the first-line remission induction therapies for severe MPA/GPA and suggest switching between the two agents when treatment response is inadequate. However, when the efficacy of RTX and/or CYC remains insufficient, a subsequent therapeutic modality with a high level of evidence has not yet been clearly validated or established [8,9].

Because circulating ANCA in peripheral blood plays a crucial role in activating primed neutrophils in MPA/GPA pathogenesis, the removal of circulating ANCA through therapeutic plasma exchange (PEX) has been theoretically considered clinically effective in alleviating disease activity and exacerbation [10,11]. Consequently, PEX has been introduced and is currently used as an adjunctive treatment for diffuse alveolar haemorrhage (DAH) and rapidly progressive glomerulonephritis (RPGN), although its indications remain limited [12,13]. However, the potential drawbacks of PEX were underscored by a large-scale clinical study that reported on increased risk of serious infections compared with its conventional benefits [14,15]. As a result, PEX was excluded from the 2021 ACR treatment recommendations and was downgraded in the 2022 EULAR guidelines, where it was suggested only as a possible therapeutic option for RPGN but not for DAH [8,9]. Nevertheless, as several studies have cautiously suggested that PEX may still be effective in specific subgroups of MPA/GPA patients, debate over its clinical usefulness in severe disease continues [16,17].

In real clinical practice, clinicians occasionally encounter patients with ANCA-negative MPA/GPA presenting with life-threatening DAH and RPGN that are refractory to RTX and CYC, even when administered alongside high-dose glucocorticoids. Furthermore, some of these patients fail to respond to additional treatment modalities such as intravenous immunoglobulin. In such urgent and refractory situations, PEX may need to be considered as an adjunctive therapeutic option, even in ANCA-negative MPA/GPA cases. To address this clinical challenge, the present study included MPA/GPA patients receiving PEX and retrospectively compared the therapeutic efficacies and preventive effects of PEX on all-cause mortality (ACM) and progression to end-stage kidney disease (ESKD) during follow-up between ANCA-positive and ANCA-negative MPA/GPA patients presenting with DAH and/or RPGN at AAV diagnosis or after diagnosis.

## 2. Materials and Methods

### 2.1. Patients

**Figure 1:**
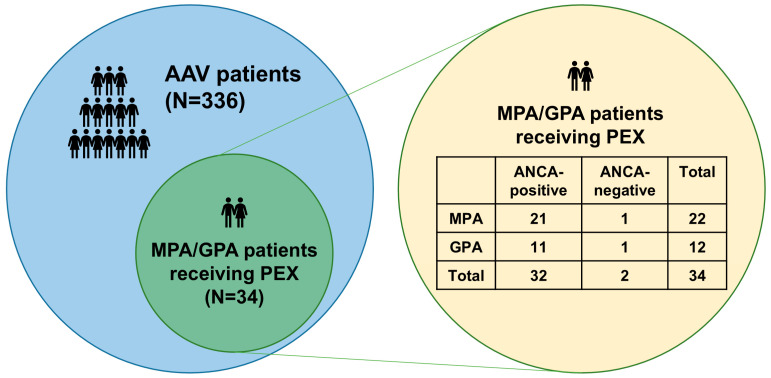
Patient selection. Of the 34 MPA/GPA patients receiving PEX, two were ANCA-negative MPA/GPA patients. AAV: ANCA-associated vasculitis; ANCA: antineutrophil cytoplasmic antibody; PEX: plasma exchange; MPA: microscopic polyangiitis; GPA: granulomatosis with polyangiitis.

We screened 336 patients enrolled in the Severance Hospital ANCA-associated VasculitidEs (SHAVE) cohort of AAV and retrospectively reviewed the medical records of the 34 selected patients who met the following inclusion criteria (Figure 1).

- Patients whose first diagnosis of MPA/GPA was made at this hospital.
- Patients whose classification of MPA/GPA was based on three established criteria: the 2007 European Medicines Agency algorithm for AAV, the 2012 revised Chapel Hill Consensus Conference nomenclature of vasculitides, and the 2022 ACR/EULAR classification criteria for MPA/GPA [1,2,5,6,7].
- Patients whose medical records were sufficiently detailed to allow collection of clinical data at AAV diagnosis and during follow-up.
- Patients for whom ANCA test results at AAV diagnosis and at the time of PEX were available [18].
- Patients who had not received immunosuppressive drugs for the treatment of MPA/GPA before diagnosis.
- Patients who had not received glucocorticoid at a prednisolone dose greater than 10 mg/day within four weeks before diagnosis.
- Patients without concurrent serious medical conditions mimicking MPA/GPA at diagnosis, such as malignancy and severe infectious disease [19,20].
- Patients who were followed up for at least three months after diagnosis.
- Patients who underwent PEX in treating MPA/GPA after diagnosis [9,12].

PEX was considered valid in this study only if it was performed a total of 5–6 times consecutively (three times a week × 2 weeks) in all patients included in the analysis.

### 2.2. Ethical Disclosure

This study was approved by the Institutional Review Board (IRB) of Severance Hospital, Seoul, Republic of Korea (IRB No. 4-2020-1071; approval date: 11 November 2016; renewal interval: every two years) and was conducted in accordance with the principles of the Declaration of Helsinki. Owing to the retrospective design of the study and the use of anonymised patient data, the requirement for written informed consent was waived.

### 2.3. ANCA Measurements and Accepted Values

Myeloperoxidase (MPO)-ANCA and proteinase 3 (PR3)-ANCA titres were measured using an enzyme immunoassay, whereas perinuclear (P)-ANCA and cytoplasmic (C)-ANCA were detected using an indirect immunofluorescent assay [18]. Both MPO-/PR3-ANCA and P-/C-ANCA results were accepted as valid ANCA findings in this study, consistent with the 2022 ACR/EULAR classification criteria [5,6,21].

### 2.4. Variables at Diagnosis

At AAV diagnosis, epidemiological data included age, sex, body mass index (BMI), and smoking history. Comorbidities such as type 2 diabetes mellitus, hypertension, and dyslipidaemia were recorded. Regarding AAV-related variables, the following were obtained: AAV subtypes, serological status, AAV-specific indices including the Birmingham Vasculitis Activity Score (BVAS; an index estimating AAV activity), the Five-Factor Score (FFS; an index predicting subsequent prognosis during follow-up), and the 36-item Short Form survey physical and mental component summary (SF-36 PCS and MCS; indices assessing functional status) [22,23,24,25]. Laboratory parameters, including erythrocyte sedimentation rate (ESR) and C-reactive protein (CRP), were also collected. In addition, the primary indication for PEX, such as DHA and RPGN that occurred at AAV diagnosis or within four weeks after diagnosis, was reviewed [16,17,26].

### 2.5. Variables During Follow-Up

During follow-up, ACM and progression to ESKD were evaluated as poor outcomes of MPA/GPA. ACM was defined as death from any cause after diagnosis, and ESKD was defined as a clinical state requiring renal replacement therapy [27]. The follow-up duration based on each poor outcome was defined as the period from AAV diagnosis to each poor outcome occurrence for patients with each poor outcome, whereas that from AAV diagnosis to the last visit for those without. The use of glucocorticoids and immunosuppressive agents after diagnosis was also assessed. PEX was considered acceptable for inclusion if it was performed for DAH or RPGN that occurred within four weeks after diagnosis. PEX was conducted immediately following the first cycle of remission induction therapy for MPA/GPA refractory to RTX and/or CYC. In addition, PEX was performed concurrently with RTX and/or CYC in patients with life-threatening MPA/GPA.

### 2.6. Statistical Analyses

All statistical analyses were conducted using IBM SPSS Statistics for Windows, version 26 (IBM Corporation, Armonk, NY, USA). Continuous variables were expressed as the median with interquartile range. Categorical variables were presented as numbers and percentages. Differences between categorical variables were analysed using the chi-square test or Fisher’s exact test, as appropriate. Meanwhile, differences between continuous variables were evaluated using the Mann–Whitney U test. Cumulative survival rates between groups were compared using the Kaplan–Meier survival test with the log-rank test. A P-value of less than 0.05 was considered statistically significant.

## 3. Results

### 3.1. Clinical Data at MPA/GPA Diagnosis

The median age of the 34 patients (22 MPA and 12 GPA) receiving PEX was 67 years (15 men and 19 women). Among them, two patients were diagnosed with ANCA-negative vasculitis (one MPA and one GPA) (Figure 1). The median BVAS, FFS, SF-36 PCS, and MCS were 18.0, 2.0, 35.8, and 27.5, respectively. Based on BVAS items, the most frequently affected major organ system was the lungs (88.2%), followed by the kidneys (85.3%). The median ESR and CRP were 63.5 mm/hr and 44.9 mg/L, respectively. The remaining laboratory data are presented in Table 1.

### 3.2. Clinical Data During Follow-Up

Of the 34 patients, 28 (82.4%) underwent PEX owing to RPGN and 6 (17.6%) due to DAH. During follow-up, 13 patients (38.2%) died after a median duration of 33.8 months following diagnosis, whereas 15 (44.1%) progressed to ESKD after a median duration of 10.5 months. All patients received glucocorticoids, and the most frequently administered immunosuppressive agent was CYC (82.4%), followed by mycophenolate mofetil (64.7%) (Table 2).

### 3.3. Comparison of Clinical Data at AAV Diagnosis and During Follow-Up Between ANCA-Positive and ANCA-Negative MPA/GPA Patients Receiving PEX

At AAV diagnosis, serum total protein and CRP levels were apparently higher in ANCA-positive MPA/GPA patients than in ANCA-negative patients; however, these differences were not clinically significant. During follow-up, there were no meaningful differences in the incidence of ACM or progression to ESKD between ANCA-positive and ANCA-negative MPA/GPA patients who underwent PEX. Furthermore, the medications administered during follow-up did not differ between the two groups (Table 3).

### 3.4. Comparison of Cumulative Survival Rates Between ANCA-Positive and ANCA-Negative MPA/GPA Patients Receiving PEX

**Figure 2:**
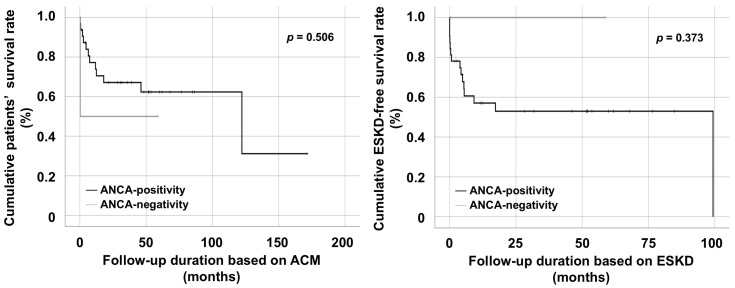
Comparison of cumulative survival rates. No significant differences in cumulative patients’ and ESKD-free survival rates were observed between ANCA-positive and ANCA-negative MPA/GPA patients. ANCA: antineutrophil cytoplasmic antibody; ACM: all-cause mortality; ESKD: end-stage kidney disease.

The cumulative patients’ survival rate of ANCA-positive MPA/GPA patients did not differ significantly from that of ANCA-negative patients. Similarly, the cumulative ESDK-free survival rate did not differ between the two groups (Figure 2).

## 4. Discussion

Given that circulating ANCA in peripheral blood directly contributes to the initiation and exacerbation of vasculitis in the pathogenesis of AAV [10], it can reasonably be inferred that PEX, which has the potential to remove circulating ANCA, may play a pivotal role in inducing and maintaining remission in ANCA-positive MPA/GPA [14,26]. In addition, when the efficacy of conventional remission induction therapy is insufficient, or when clinical manifestations are so severe that it is difficult to await the full therapeutic responses, PEX may need to be considered even in ANCA-negative MPA/GPA patients as an adjunctive therapeutic modality in real clinical practice [12,13]. To address this clinical challenge, the present study included MPA/GPA patients receiving PEX and retrospectively compared the therapeutic efficacies and preventive effects of PEX on ACM and ESKD between ANCA-positive and ANCA-negative MPA/GPA patients presenting with DAH and/or RPGN. Several findings were obtained. First, among the 336 patients with AAV, 34 MPA/GPA patients underwent PEX. Second, of these 34 patients, PEX was performed owing to DAH and RPGN in 17.6% and 82.4%, respectively. Third, among the patients who underwent PEX, two were diagnosed with ANCA-negative vasculitis (one MPA and one GPA). Fourth, during follow-up, 13 patients died and 15 progressed to ESKD. Fifth, no clinically significant differences in variables at AAV diagnosis and during follow-up were observed between ANCA-positive and ANCA-negative MPA/GPA patients. Finally, cumulative patients’ and ESKD-free survival rates did not differ between the two groups.

Given the clinical role of PEX in alleviating disease activity and improving poor outcomes of AAV by removing circulating ANCA in peripheral blood, it might initially seem clinically unreasonable to consider PEX in treating ANCA-negative MPA/GPA. However, the present study demonstrated that PEX yielded non-inferior results in ANCA-negative MPA/GPA patients compared with ANCA-positive MPA/GPA patients despite the limited sample size. Several inferences were made regarding the potential mechanism of PEX in ANCA-negative MPA/GPA patients. The first inference was that circulating ANCA may have been present but undetectable owing to titres being below the threshold of detection [18,28]. This hypothesis has two implications: first, that spontaneous fluctuations in ANCA concentration may result in temporary seronegativity, and second, that the sensitivity of current immunoassay techniques may be insufficient to detect very low antibody levels. The second inference was that ANCA-negativity might result from antigenic epitope diversity, implying the presence of ANCA variants that fail to recognise the specific epitopes used in standard detection assays [29]. Conversely, the third inference was that even in the absence of circulating ANCA, PEX may exert therapeutic benefits in MPA/GPA by removing various inflammatory mediators involved in AAV pathophysiology [30,31]. Considering the balance between the therapeutic benefits and potential complications of PEX, the last hypothesis could provide a rationale for its use as a therapeutic option in severe and refractory ANCA-negative MPA/GPA in clinical practice [32].

Given the result in the comparative analysis of the efficacy of PEX between ANCA-positive and ANCA-negative MPA/GPA, particularly its preventive effect on poor outcomes, to validate the robustness of this result, it would be adequate to conduct a further subgroup analysis. Accordingly, patients were divided into four subgroups based on the following factors: (i) DAH; (ii) RPGN; (iii) MPA; and (iv) GPA. The Kaplan–Meier survival analyses were planned to compare the preventive effects of PEX on ACM and ESKD occurrence. However, statistical analysis was not feasible because each subgroup contained only one patient. Instead, the preventive effect of PEX on poor outcomes during follow-up was compared between the two groups, irrespective of ANCA positivity. First, when patients were stratified according to DAH or RPGN, no significant difference in cumulative patients’ survival rates was observed between the two subgroups (Supplementary Figure S1). Comparative analysis of cumulative ESKD-free survival rates was not performed because ESKD occurrence is intrinsically associated with RPGN. Second, when patients were divided according to AAV subtype, no significant differences were found in cumulative patients’ and ESKD-free survival rates between the two groups (Supplementary Figure S2). Therefore, although this represents a preliminary finding based on a limited number of patients, it may be inferred that in ANCA-negative MPA/GPA, the presence of DAH or RPGN and the AAV subtype may not need to be decisive factors when considering PEX treatment.

This study showed the median age of 67.0 years, BMI of 22.1 kg/m^2^, and dyslipidaemia prevalence of 23.5%, which were found to be lower than the average BMI and dyslipidaemia prevalence for Korean individuals aged 65–75 [33,34,35]. In general, chronic inflammation is known to be correlated with an increase in BMI and an elevated prevalence of dyslipidaemia [36]. Since AAV is a representative chronic inflammatory disease, we expected the BMI value and dyslipidaemia prevalence to be elevated in the study subjects, but the results were the opposite. We hypothesise that patients with severe/refractory MPA/GPA requiring PEX might have exhibited a pattern of low BMI and low dyslipidaemia prevalence associated with wasting cachexia rather than a pattern of inflammation-induced obesity and hyperlipidaemia. Additionally, it should be emphasised that the impact of obesity or dyslipidaemia on the prognosis of AAV through metabolic dysfunction was not significant in this study.

This study has the advantage of being the first to demonstrate the clinical feasibility of PEX for the treatment of severe ANCA-negative MPA and GPA refractory to the initial re-mission-induction therapy as a pilot study. Meanwhile, this study has several limitations. The most critical limitation is the small number of MPA/GPA patients receiving PEX. Since increasing the number of patients will augment the validity of the results, but cases where PEX is used for severe and refractory ANCA-negative MPA/GPA are rare in real clinical settings, it would be more practical to plan future studies with a larger number of ANCA-negative patients. Another limitation is that the study was retrospectively conducted at a single centre. Particularly, it seemed impossible to record every drug prescribed by other hospitals or other departments. For this reason, we had no choice but to limit our investigation to glucocorticoids and immunosuppressive drugs. Also, this limitation prevented us from demonstrating the fluctuation of circulating ANCA levels in blood samples of ANCA-negative patients or investigating epitope diversity using immunoassays designed with various fixed epitopes. This discouraged us from providing supporting evidence for our two inferences. Nevertheless, as a pilot study, it holds clinical significance because it provides preliminary evidence suggesting that PEX may be considered in ANCA-negative MPA/GPA patients who are refractory to the first-line remission induction therapy. Additionally, despite the close association between mortality and cardiac dysfunction, echocardiography or biomarkers estimating or predicting the deterioration in heart function, particularly heart failure with preserved ejection fraction, were not performed (or recorded) in all patients. A prospective, multicentre study including a larger number of ANCA-negative MPA/GPA patients treated with PEX for DAH and RPGN is warranted to further clarify the therapeutic role of PEX in managing severe and refractory ANCA-negative MPA/GPA.

## 5. Conclusions

In conclusion, this study was the first pilot study to demonstrate the clinical feasibility of PEX for the treatment of severe ANCA-negative MPA and GPA refractory to the initial remission induction therapy.

## Figures and Tables

**Table 1:** Clinical data at AAV diagnosis among MPA/GPA patients receiving PEX (N = 34).

Variables	Values
At AAV diagnosis	
Demographic data	
Age (years)	67.0 (55.5–73.5)
Male sex (N, (%))	15 (44.1)
Female sex (N, (%))	19 (55.9)
BMI (kg/m^2^)	22.1 (19.9–24.8)
Ex-smoker	1 (2.9)
Comorbidities (N, (%))	
T2DM	6 (17.6)
Hypertension	15 (44.1)
Dyslipidaemia	8 (23.5)
AAV subtype (N, (%))	
MPA	22 (64.7)
GPA	12 (35.3)
ANCA type and positivity (N, (%))	
MPO-ANCA (or P-ANCA) positivity	25 (73.5)
PR3-ANCA (or C-ANCA) positivity	7 (20.6)
Both ANCAs	0 (0)
No ANCA	2 (5.9)
AAV-specific indices	
BVAS	18.0 (12.0–23.0)
FFS	2.0 (1.0–2.0)
SF-36 PCS	35.8 (18.4–45.3)
SF-36 MCS	37.5 (25.2–54.3)
Major organ involvements based on BVAS items (N, (%))	
General symptoms	19 (55.9)
Skin	3 (8.8)
Mucosa and eyes	1 (2.9)
Ears, nose, and throat	15 (44.1)
Lungs	30 (88.2)
Heart	6 (17.6)
Abdomen	1 (2.9)
Kidneys	29 (85.3)
CNS/PNS	6 (17.6)
Laboratory results	
White blood cell count (/mm^3^)	11,365.0 (7002.5–14,155.0)
Haemoglobin (g/dL)	9.1 (8.2–10.7)
Platelet count (×1000/mm^3^)	262.5 (204.0–390.0)
Fasting glucose (mg/dL)	111.5 (91.0–142.3)
Blood urea nitrogen (mg/dL)	36.6 (21.1–55.3)
Serum creatinine (mg/dL)	2.5 (1.2–5.4)
Serum total protein (g/dL)	6.0 (5.4–6.7)
Serum albumin (g/dL)	3.2 (2.8–3.6)
ESR (mm/hr)	63.5 (12.5–115.3)
CRP (mg/L)	44.9 (7.2–95.4)

Values are expressed as a median (25–75 percentile) or N (%). MPA: microscopic polyangiitis; GPA: granulomatosis with polyangiitis; PEX: plasma exchange; BMI: body mass index; T2DM: type 2 diabetes mellitus; MPO: myeloperoxidase; ANCA: antineutrophil cytoplasmic antibody; P: perinuclear; PR3: proteinase 3; C: cytoplasmic; BVAS: the Birmingham vasculitis activity score; FFS: the five-factor score; SF-36: 36-item short form survey; PCS: physical component summary; MCS: mental component summary; CNS: central nervous system; PNS: peripheral nervous system; ESR: erythrocyte sedimentation rate; CRP: C-reactive protein.

**Table 2:** Clinical data during follow-up among MPA/GPA patients receiving PEX (N = 34).

Variables	Values
Reasons for considering PEX (N, (%))	
DAH	6 (17.6)
RPGN	28 (82.4)
Poor outcomes	
ACM (N, (%))	13 (38.2)
Follow-up duration based on ACM (months)	33.8 (6.0–59.1)
ESKD (N, (%))	15 (44.1)
Follow-up duration based on ESKD (months)	10.5 (1.7–52.4)
Medications administered (N, (%))	
GC	34 (100)
RTX	15 (44.1)
CYC	28 (82.4)
MMF	22 (64.7)
AZA	16 (47.1)
TAC	4 (11.8)
MTX	3 (8.8)

Values are expressed as a median (25–75 percentile) or N (%). DAH: diffuse alveolar haemorrhage; RPGN: rapidly progressive glomerulonephritis; MPA: microscopic polyangiitis; GPA: granulomatosis with polyangiitis; PEX: plasma exchange; ACM: all-cause mortality; ESKD: end-stage kidney disease; GC: glucocorticoids; RTX: rituximab; CYC: cyclophosphamide; MMF: mycophenolate mofetil; AZA: azathioprine; TAC: tacrolimus; MTX: methotrexate.

**Table 3:** Comparison of variables between ANCA-positive and ANCA-negative MPA/GPA patients receiving PEX (N = 34).

Variables	ANCA-Positive MPA/GPA Patients (N = 32)	ANCA-Negative MPA/GPA Patients (N = 2)	*p*-Value
At AAV diagnosis			
Demographic data			
Age (years)	69.0 (12.5)	61.0 (N/A)	0.913
Male sex (N, (%))	14 (43.8)	1 (50.0)	1.000
Female sex (N, (%))	18 (56.3)	1 (50.0)	1.000
BMI (kg/m^2^)	21.8 (4.2)	25.6 (N/A)	0.187
Ex-smoker	1 (3.1)	0 (0)	1.000
Comorbidities (N, (%))			
T2DM	6 (18.8)	0 (0)	1.000
Hypertension	15 (46.9)	0 (0)	0.492
Dyslipidaemia	8 (25.0)	0 (0)	1.000
AAV subtype (N, (%))			1.000
MPA	21 (65.6)	1 (50.0)	
GPA	11 (34.4)	1 (50.0)	
ANCA type and positivity (N, (%))			
MPO-ANCA (or P-ANCA) positivity	25 (78.1)	0 (0)	0.064
PR3-ANCA (or C-ANCA) positivity	7 (21.9)	0 (0)	1.000
AAV-specific indices			
BVAS	18.0 (8.0)	19.0 (N/A)	0.741
FFS	2.0 (1.0)	2.0 (0)	0.618
SF-36 PCS	36.3 (25.3)	20.0 (N/A)	0.197
SF-36 MCS	36.9 (29.6)	28.8 (N/A)	0.533
Major organ involvements based on BVAS items (N, (%))			
General symptoms	18 (56.3)	1 (50.0)	1.000
Skin	2 (6.3)	1 (50.0)	0.171
Mucosa and eyes	1 (3.1)	0 (0)	1.000
Ears, nose, and throat	14 (43.8)	1 (50.0)	1.000
Lungs	28 (87.5)	2 (100)	1.000
Heart	6 (18.8)	0 (0)	1.000
Abdomen	1 (3.1)	0 (0)	1.000
Kidneys	27 (84.4)	2 (100)	1.000
CNS/PNS	5 (15.6)	1 (50.0)	0.326
Laboratory results			
White blood cell count (/mm^3^)	11,290.0 (7010.0)	13,070.0 (N/A)	0.421
Haemoglobin (g/dL)	8.6 (2.6)	9.8 (N/A)	0.442
Platelet count (×1000/mm^3^)	259.0 (199.5)	262.5 (N/A)	1.000
Fasting glucose (mg/dL)	101.0 (52.0)	120.5 (N/A)	0.971
Blood urea nitrogen (mg/dL)	40.7 (38.7)	22.4 (N/A)	0.213
Serum creatinine (mg/dL)	2.5 (4.4)	0.8 (N/A)	0.073
Serum total protein (g/dL)	6.0 (1.4)	4.8 (N/A)	0.037
Serum albumin (g/dL)	3.1 (1.1)	3.2 (N/A)	0.883
ESR (mm/hr)	69.0 (106.0)	61.0 (N/A)	0.875
CRP (mg/L)	37.7 (84.5)	1.6 (N/A)	0.044
Reasons for considering PEX (N, (%))			0.326
DAH	5 (15.6)	1 (50.0)	
RPGN	27 (84.4)	1 (50.0)	
During AAV follow-up			
Poor outcomes			
ACM (N, (%))	12 (37.5)	1 (50.0)	1.000
Follow-up duration based on ACM (months)	35.7 (47.3)	29.6 (N/A)	0.583
ESKD (N, (%))	15 (46.9)	0 (0)	0.492
Follow-up duration based on ESKD (months)	12.5 (48.4)	29.6 (N/A)	0.942
Medications administered (N, (%))			
GC	32 (100)	2 (100)	N/A
RTX	15 (46.9)	0 (0)	0.492
CYC	26 (81.3)	2 (100)	1.000
MMF	20 (62.5)	2 (100)	0.529
AZA	15 (46.9)	1 (50.0)	1.000
TAC	3 (9.4)	1 (50.0)	0.225
MTX	2 (6.3)	1 (50.0)	0.171

Values are expressed as a median (interquartile range) or N (%). ANCA: antineutrophil cytoplasmic antibody; MPA: microscopic polyangiitis; GPA: granulomatosis with polyangiitis; PEX: plasma exchange; N/A: not applicable; BMI: body mass index; T2DM: type 2 diabetes mellitus; MPO: myeloperoxidase; P: perinuclear; PR3: proteinase 3; C: cytoplasmic; BVAS: the Birmingham vasculitis activity score; FFS: the five-factor score; SF-36: 36-item short form survey; PCS: physical component summary; MCS: mental component summary; eVDI: early vasculitis damage index; CNS: central nervous system; PNS: peripheral nervous system; ESR: erythrocyte sedimentation rate; CRP: C-reactive protein; DAH: diffuse alveolar haemorrhage; RPGN: rapidly progressive glomerulonephritis; ACM: all-cause mortality; ESKD: end-stage kidney disease; GC: glucocorticoids; RTX: rituximab; CYC: cyclophosphamide; MMF: mycophenolate mofetil; AZA: azathioprine; TAC: tacrolimus; MTX: methotrexate.

## Data Availability

The dataset collected and/or analysed in the present study is available on request from the corresponding author.

## Supplementary Materials

The following supporting information can be downloaded at https://www.mdpi.com/article/10.3390/medicina61122184/s1, Figure S1: Comparison of cumulative patients’ survival rates between patients with DAH and those with RPGN. Figure S2: Comparison of cumulative patients’ and ESKD-free survival rates between MPA and GPA patients.

## References

[1] Jennette J.C., Falk R.J., Bacon P.A., Basu N., Cid M.C., Ferrario F., Flores-Suarez L.F., Gross W.L., Guillevin L., Hagen E.C. (2013). 2012 revised International Chapel Hill Consensus Conference Nomenclature of Vasculitides. Arthritis Rheum..

[2] Watts R., Lane S., Hanslik T., Hauser T., Hellmich B., Koldingsnes W., Mahr A., Segelmark M., Cohen-Tervaert J.W., Scott D. (2007). Development and validation of a consensus methodology for the classification of the ANCA-associated vasculitides and polyarteritis nodosa for epidemiological studies. Ann. Rheum. Dis..

[3] Triggianese P., D’Antonio A., Nesi C., Kroegler B., Di Marino M., Conigliaro P., Modica S., Greco E., Nucci C., Bergamini A. (2023). Subclinical microvascular changes in ANCA-vasculitides: The role of optical coherence tomography angiography and nailfold capillaroscopy in the detection of dis-ease-related damage. Orphanet J. Rare Dis..

[4] Screm G., Gandin I., Mondini L., Cifaldi R., Confalonieri P., Bozzi C., Salton F., Bandini G., Monteleone G., Hughes M. (2025). Evaluation of Nailfold Capillaroscopy as a Novel Tool in the Assessment of Eosinophilic Granulomatosis with Polyangiitis. J. Clin. Med..

[5] Suppiah R., Robson J.C., Grayson P.C., Ponte C., Craven A., Khalid S., Judge A., Hutchings A., Merkel P.A., Luqmani R.A. (2022). 2022 American College of Rheumatology/European Alliance of Associations for Rheumatology classification criteria for microscopic polyangiitis. Ann. Rheum. Dis..

[6] Robson J.C., Grayson P.C., Ponte C., Suppiah R., Craven A., Judge A., Khalid S., Hutchings A., Luqmani R.A., A Watts R. (2022). 2022 American College of Rheumatology/European Alliance of Associations for Rheumatology classification criteria for granulomatosis with polyangiitis. Ann. Rheum. Dis..

[7] Pyo J.Y., Lee L.E., Park Y.B., Lee S.W. (2023). Comparison of the 2022 ACR/EULAR Classification Criteria for Antineutrophil Cytoplasmic Antibody-Associated Vasculitis with Previous Criteria. Yonsei Med. J..

[8] Chung S.A., Langford C.A., Maz M., Abril A., Gorelik M., Guyatt G., Archer A.M., Conn D.L., Full K.A., Grayson P.C. (2021). 2021 American College of Rheumatology/Vasculitis Foundation Guideline for the Management of Antineutrophil Cytoplasmic Antibody-Associated Vasculitis. Arthritis Rheumatol..

[9] Hellmich B., Sanchez-Alamo B., Schirmer J.H., Berti A., Blockmans D., Cid M.C., Holle J.U., Hollinger N., Karadag O., Kronbichler A. (2024). EULAR recommendations for the management of ANCA-associated vasculitis: 2022 update. Ann. Rheum. Dis..

[10] Kitching A.R., Anders H.J., Basu N., Brouwer E., Gordon J., Jayne D.R., Kullman J., Lyons P.A., Merkel P.A., Savage C.O. (2020). ANCA-associated vasculitis. Nat. Rev. Dis. Primers.

[11] van der Geest K.S.M., Brouwer E., Sanders J.S., Sandovici M., Bos N.A., Boots A.M.H., Abdulahad W.H., Stegeman C.A., Kallenberg C.G.M., Heeringa P. (2018). Towards precision medicine in ANCA-associated vasculitis. Rheumatology.

[12] Yates M., Watts R.A., Bajema I.M., Cid M.C., Crestani B., Hauser T., Hellmich B., Holle J.U., Laudien M., Little M.A. (2016). EULAR/ERA-EDTA recommendations for the management of ANCA-associated vasculitis. Ann. Rheum. Dis..

[13] Tsiakas S., Marinaki S., Lionaki S., Boletis J. (2021). Plasma Exchange in ANCA-Associated Vasculitis: A Narrative Review. J. Clin. Med..

[14] Walsh M., Merkel P.A., Peh C.A., Szpirt W.M., Puéchal X., Fujimoto S., Hawley C.M., Khalidi N., Floßmann O., Wald R. (2020). Plasma Exchange and Glucocorticoids in Severe ANCA-Associated Vasculitis. N. Engl. J. Med..

[15] Walsh M., Collister D., Zeng L., Merkel P.A., Pusey C.D., Guyatt G., Peh C.A., Szpirt W., Ito-Hara T., Jayne D.R.W. (2022). The effects of plasma exchange in patients with ANCA-associated vasculitis: An updated systematic review and meta-analysis. BMJ.

[16] Esposito P., Cipriani L., Viazzi F. (2020). Plasma Exchange and Glucocorticoids in Severe ANCA-Associated Vasculitis. N. Engl. J. Med..

[17] Odler B., Riedl R., Geetha D., Szpirt W.M., Hawley C., Uchida L., Wallace Z.S., Walters G., Muso E., Tesar V. (2025). The effects of plasma exchange and glucocorticoids on early kidney function among patients with ANCA-associated vasculitis in the PEXIVAS trial. Kidney Int..

[18] Bossuyt X., Cohen Tervaert J.W., Arimura Y., Blockmans D., Flores-Suárez L.F., Guillevin L., Hellmich B., Jayne D., Jennette J.C., Kallenberg C.G. (2017). Position paper: Revised 2017 international consensus on testing of ANCAs in granulomatosis with polyangiitis and microscopic polyangiitis. Nat. Rev. Rheumatol..

[19] Phatak S., Aggarwal A., Agarwal V., Lawrence A., Misra R. (2017). Antineutrophil cytoplasmic antibody (ANCA) testing: Audit from a clinical immunology laboratory. Int. J. Rheum. Dis..

[20] Moiseev S., Cohen Tervaert J.W., Arimura Y., Bogdanos D.P., Csernok E., Damoiseaux J., Ferrante M., Flores-Suárez L.F., Fritzler M.J., Invernizzi P. (2020). 2020 international consensus on ANCA testing beyond systemic vasculitis. Autoimmun. Rev..

[21] McAdoo S.P., Medjeral-Thomas N., Gopaluni S., Tanna A., Mansfield N., Galliford J., Griffith M., Levy J., Cairns T.D., Jayne D. (2019). Long-term follow-up of a combined rituximab and cyclophosphamide regimen in renal anti-neutrophil cytoplasm antibody-associated vasculitis. Nephrol. Dial. Transplant..

[22] Mukhtyar C., Lee R., Brown D., Carruthers D., Dasgupta B., Dubey S., Flossmann O., Hall C., Hollywood J., Jayne D. (2009). Modification and validation of the Birmingham Vasculitis Activity Score (version 3). Ann. Rheum. Dis..

[23] Guillevin L., Pagnoux C., Seror R., Mahr A., Mouthon L., Toumelin P.L. (2011). French Vasculitis Study Group. The Five-Factor Score revisited: Assessment of prognoses of systemic necrotizing vasculitides based on the French Vasculitis Study Group (FVSG) cohort. Medicine.

[24] Han C.W., Lee E.J., Iwaya T., Kataoka H., Kohzuki M. (2004). Development of the Korean version of Short-Form 36-Item Health Survey: Health related QOL of healthy elderly people and elderly patients in Korea. Tohoku J. Exp. Med..

[25] Mukhtyar C., Flossmann O., Hellmich B., Bacon P., Cid M., Cohen-Tervaert J.W., Gross W.L., Guillevin L., Jayne D., Mahr A. (2008). Outcomes from studies of antineutrophil cytoplasm antibody associated vasculitis: A systematic review by the European League Against Rheumatism systemic vasculitis task force. Ann. Rheum. Dis..

[26] Fussner L.A., Flores-Suárez L.F., Cartin-Ceba R., Specks U., Cox P.G., Jayne D.R.W., Gross W.L., Guillevin L., Jayne D., Mahr A. (2024). Alveolar Hemorrhage in Antineutrophil Cytoplasmic Antibody-Associated Vasculitis: Results of an International Randomized Controlled Trial (PEXIVAS). Am. J. Respir. Crit. Care Med..

[27] Webster A.C., Nagler E.V., Morton R.L., Masson P. (2017). Chronic Kidney Disease. Lancet.

[28] Rao D.A., Wei K., Merola J.F., O’Brien W.R., Takvorian S.U., Dellaripa P.F., Schur P.H. (2015). Myeloperoxidase-antineutrophil Cytoplasmic Antibodies (MPO-ANCA) and Proteinase 3-ANCA without Immunofluorescent ANCA Found by Routine Clinical Testing. J. Rheumatol..

[29] Roth A.J., Ooi J.D., Hess J.J., van Timmeren M.M., Berg E.A., Poulton C.E., McGregor J., Burkart M., Hogan S.L., Hu Y. (2013). Epitope specificity determines pathogenicity and detectability in ANCA-associated vasculitis. J. Clin. Investig..

[30] Sauer A., Stahl K., Seeliger B., Wendel-Garcia P.D., Lehmann F., Schmidt J.J., Schmidt B.M.W., Welte T., Peukert K., Wild L. (2025). The effect of therapeutic plasma exchange on the inflammatory response in septic shock: A secondary analysis of the EXCHANGE-1 trial. Intensive Care Med. Exp..

[31] Rony R.M.I.K., Shokrani A., Malhi N.K., Hussey D., Mooney R., Chen Z.B., Scott T., Han H., Moore J., Liu J. (2025). Therapeutic Plasma Exchange: Current and Emerging Applications to Mitigate Cellular Signaling in Disease. Biomolecules.

[32] Cervantes C.E., Bloch E.M., Sperati C.J. (2023). Therapeutic Plasma Exchange: Core Curriculum 2023. Am. J. Kidney Dis..

[33] Yi S.W., Ohrr H., Shin S.A., Yi J.J. (2015). Sex-age-specific association of body mass index with all-cause mortality among 12.8 million Korean adults: A prospective cohort study. Int. J. Epidemiol..

[34] Jeong S.M., Jung J.H., Yang Y.S., Kim W., Cho I.Y., Lee Y.B., Park K.-Y., Nam G.E., Han K., Taskforce Team of the Obesity Fact Sheet of the Korean Society for the Study of Obesity (2024). 2023 Obesity Fact Sheet: Prevalence of Obesity and Abdominal Obesity in Adults, Adolescents, and Children in Korea from 2012 to 2021. J. Obes. Metab. Syndr..

[35] Kwon O., Lee S.Y., Kim B., Han K., Ahn J. (2025). On behalf of the Committee of Public Relations of the Korean Society of Lipid and Atherosclerosis. Dyslipidemia Fact Sheet in South Korea, 2024. J. Lipid. Atheroscler..

[36] McDade T.W., Meyer J.M., Koning S.M., Harris K.M. (2021). Body mass and the epidemic of chronic inflammation in early mid-adulthood. Soc. Sci. Med..

