# Supplementary figures and images for: Plasma Exchange as an Adjunctive Therapeutic Option for Severe and Refractory Antineutrophil Cytoplasmic Antibody-Negative Microscopic Polyangiitis and Granulomatosis with Polyangiitis
Source: Medicina (Kaunas). 2025 Dec 9;61(12):2184. doi: 10.3390/medicina61122184 (PMC12735260; doi:10.3390/medicina61122184)

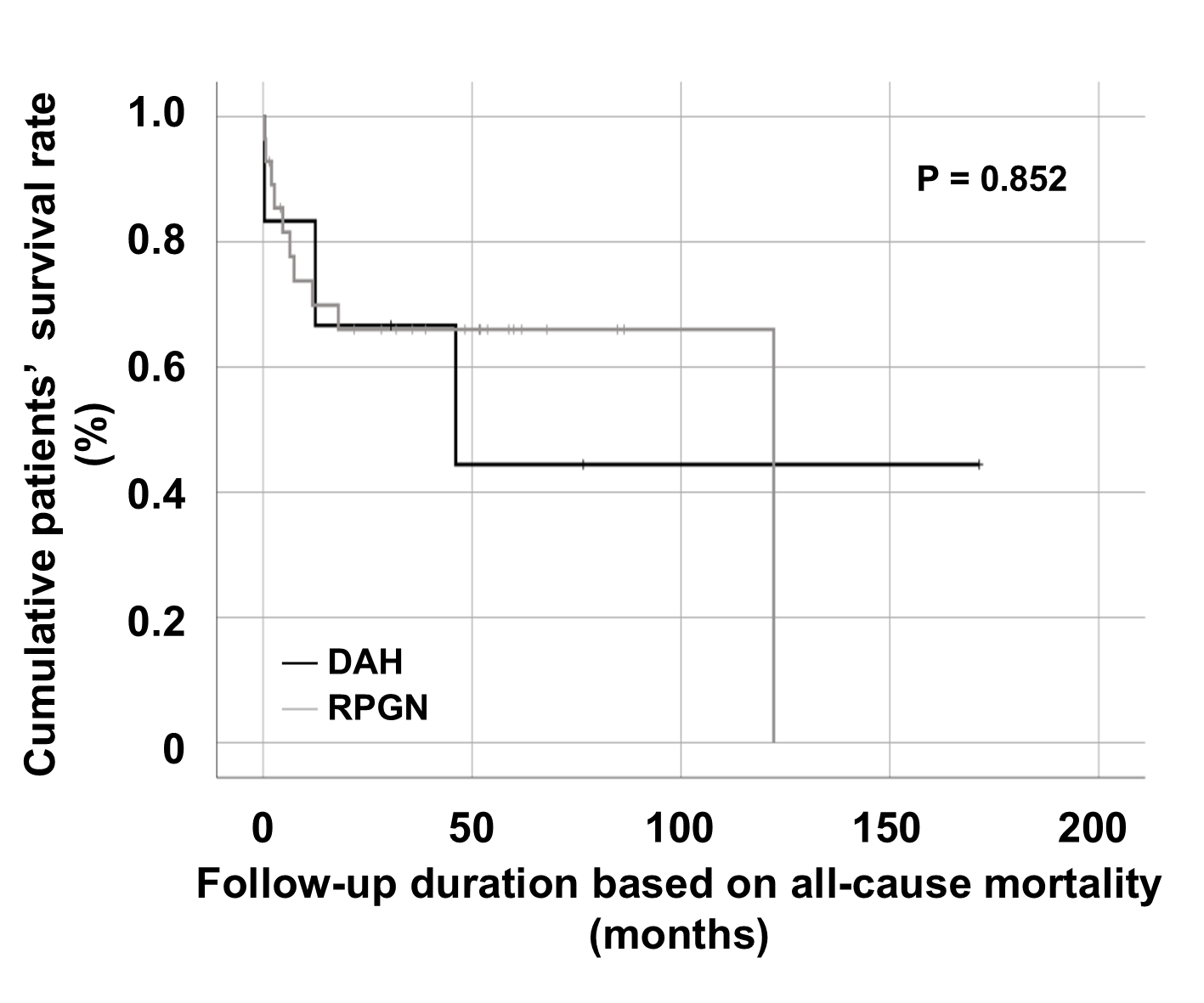

Supplement: Supplementary file 1 [file medicina-61-02184-s001.zip › SUPPLEMENTARY FIGURE1.tif]

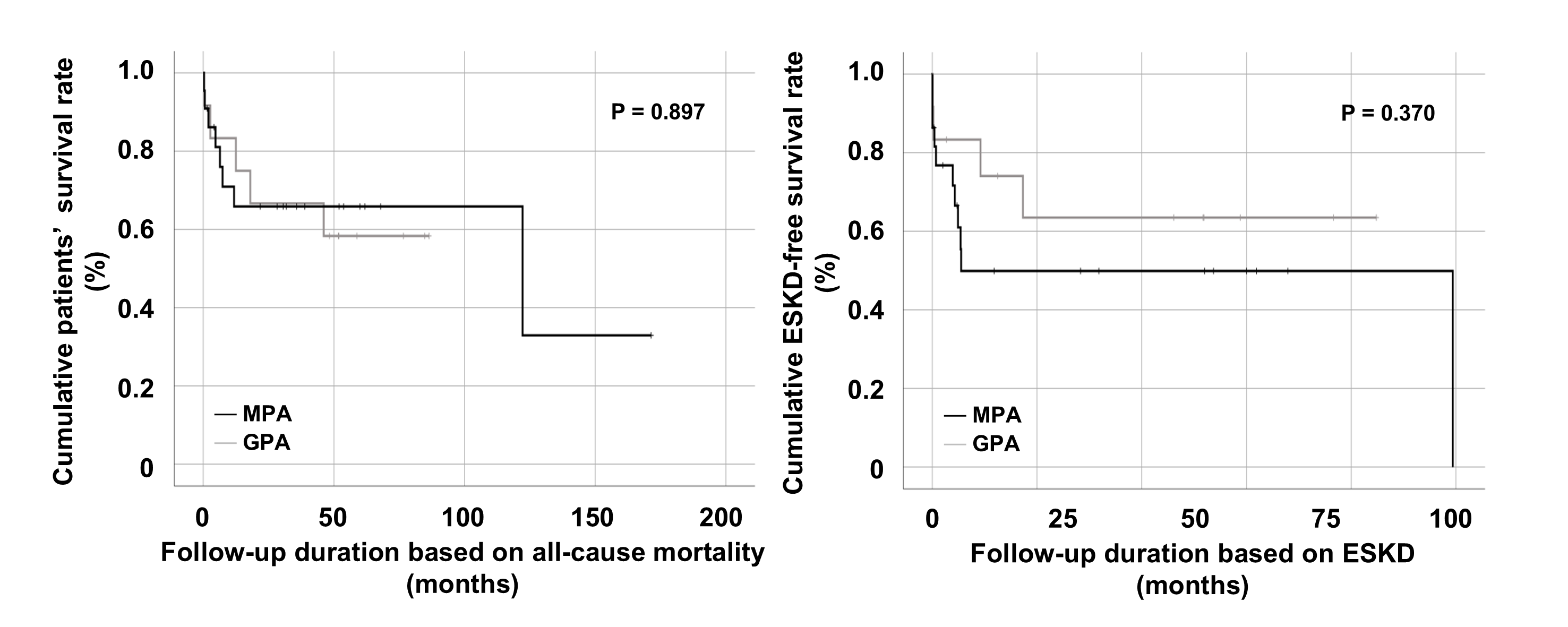

Supplement: Supplementary file 1 [file medicina-61-02184-s001.zip › SUPPLEMENTARY FIGURE2.tif]
